# Life-course neighbourhood deprivation and brain structure in older adults: the Lothian Birth Cohort 1936

**DOI:** 10.1038/s41380-024-02591-9

**Published:** 2024-05-21

**Authors:** Gergő Baranyi, Colin R. Buchanan, Eleanor L. S. Conole, Ellen V. Backhouse, Susana Muñoz Maniega, María del C. Valdés Hernández, Mark E. Bastin, Joanna Wardlaw, Ian J. Deary, Simon R. Cox, Jamie Pearce

**Affiliations:** 1 Centre for Research on Environment, Society and Health, School of GeoSciences, The University of Edinburgh, Edinburgh, UK; 2 Lothian Birth Cohorts, Department of Psychology, The University of Edinburgh, Edinburgh, UK; 3 Scottish Imaging Network, A Platform for Scientific Excellence (SINAPSE) Collaboration, Edinburgh, UK; 4 Centre for Clinical Brain Sciences (CCBS), The University of Edinburgh, Edinburgh, UK; 5 UK Dementia Research Institute Centre at the University of Edinburgh, Edinburgh, UK; 6 Edinburgh Imaging, The University of Edinburgh, Edinburgh, UK

**Keywords:** Biomarkers, Neuroscience, Psychology

## Abstract

Neighbourhood disadvantage may be associated with brain health but the importance of exposure at different stages of the life course is poorly understood. Utilising the Lothian Birth Cohort 1936, we explored the relationship between residential neighbourhood deprivation from birth to late adulthood, and global and local neuroimaging measures at age 73. A total of 689 participants had at least one valid brain measures (53% male); to maximise the sample size structural equation models with full information maximum likelihood were conducted. Residing in disadvantaged neighbourhoods in mid- to late adulthood was associated with smaller total brain (*β* = −0.06; SE = 0.02; sample size[*N*] = 658; number of pairwise complete observations[*n*]=390), grey matter (*β* = −0.11; SE = 0.03; *N* = 658; *n* = 390), and normal-appearing white matter volumes (*β* = −0.07; SE = 0.03; *N* = 658; *n* = 390), thinner cortex (*β* = −0.14; SE = 0.06; *N* = 636; *n* = 379), and lower general white matter fractional anisotropy (*β* = −0.19; SE = 0.06; *N* = 665; *n* = 388). We also found some evidence on the accumulating impact of neighbourhood deprivation from birth to late adulthood on age 73 total brain (*β* = −0.06; SE = 0.02; *N* = 658; *n* = 276) and grey matter volumes (*β* = −0.10; SE = 0.04; *N* = 658; *n* = 276). Local analysis identified affected focal cortical areas and specific white matter tracts. Among individuals belonging to lower social classes, the brain-neighbourhood associations were particularly strong, with the impact of neighbourhood deprivation on total brain and grey matter volumes, and general white matter fractional anisotropy accumulating across the life course. Our findings suggest that living in deprived neighbourhoods across the life course, but especially in mid- to late adulthood, is associated with adverse brain morphologies, with lower social class amplifying the vulnerability.

## Introduction

Ageing-related cognitive decline is highly prevalent with one in four community-dwelling adults over the age of 60 experiencing noticeable decline in cognitive functions [1]. Cognitive decline is a key dimension of ageing; it heralds dementia, illness, and death [2, 3]. Brain structural measures are a well-established set of cognitive ageing biomarkers [4]. It is critical to understand risk factors of structural brain differences, potential mechanisms, and biomarkers to inform rational bases for early interventions and to identify those at greatest risk of cognitive decline and dementia. Brain structure and functioning in later life has been linked to more proximal social determinants of health across the life course, including adversities [5, 6] and individual socioeconomic position [7–9]; however, the long-term impact of distal factors in the life course, such as neighbourhood deprivation, are less understood. Neighbourhood disadvantage is an important modifiable predictor of old age health [10] and cognition [11] with effects distinct from individual socioeconomic factors.

Residing in disadvantaged areas might influence brain morphology through multiple interrelated ways and the underlying processes may differ across the life course. Socioeconomically disadvantaged areas are likely to suffer from poorer housing conditions [12], higher crime and violence [13], have lower provision of high quality green space [14], and residents are more likely to be exposed to higher levels of contaminants (e.g., air pollution [15]), and to health-damaging commodities and services, such as alcohol, fast food and tobacco outlets [16]; these environmental features, in turn, can associate directly with brain morphology and function (e.g., green space [17], air pollution [18]). In childhood, the quality of local schools may be pertinent, as might the availability of neighbourhood resources and amenities (e.g., community and cultural centres) in late adulthood. Research shows that children exposed to stressors in their social environment experience activated hypothalamic-pituitary-adrenal axis which leads to long-term dysregulation and changes in the brain [19, 20]; investigated pathways in adulthood include inflammatory [21, 22], neuroendocrine [23] and cardiovascular [24] mechanisms.

Existing research on neighbourhood deprivation and brain health in late adulthood is limited by cross-sectional or short-term longitudinal study designs that offer only a snapshot of current environmental conditions [23–26]. Given that neighbourhood exposures, especially during sensitive periods of brain development, might have very long-term impacts on brain health, it is important to account for exposures across the whole life, though such data are rare. Applying the life-course approach (i.e., examining the life-course impact of social and physical exposures on later health and diseases risk [27]) has the potential to partially overcome methodological biases [11], and it has been applied to explore individual-level risk factors of brain health among older adults [7–9]. Still, reconstructing objectively measured historical neighbourhood exposures over several decades remains a significant challenge due to the lack of consistently measured neighbourhood-level data and residential history covering the entire life course [28, 29].

The present life-course study investigated whether living in deprived neighbourhoods from birth onwards was associated with global and local brain morphology among older adults. Eight global brain measures, likely associated with cognitive function [30], were selected as outcomes. Six related to white and grey matter macrostructure (total brain, grey matter, normal-appearing white matter and white matter hyperintensity volumes, cortical surface area, and mean cortical thickness). Two commonly used indicators of white matter microstructure included fractional anisotropy (i.e., directional coherence of water molecule diffusion), and mean diffusivity (i.e., magnitude of water molecule diffusion); these biomarkers provide information on the microstructural environment within the brain’s white matter, for example, including but not limited to declining axonal integrity and myelination with increasing age [31]. Such structural changes lead to less directionally coherent water molecular diffusion, and thereby decreased fractional anisotropy and increased mean diffusivity. Vertex-wise and white matter tract-specific analyses identified areas of interest. We tested four exposure models to explain individual differences in grey and white matter macro- and microstructure at age 73 years: neighbourhood deprivation in three sensitive periods (childhood, young adulthood, and mid- to late adulthood), and accumulated life-course neighbourhood deprivation. Little is known about whether some population groups show stronger associations between neighbourhood deprivation and brain health [24]; therefore, we explored effect modification by sex, apolipoprotein E (*APOE*) ε4 allele status, and individual-level social disadvantage in childhood and adulthood.

## Methods

### Study participants

Data were drawn from the LBC1936, a longitudinal study of relatively healthy older adults born in 1936. LBC1936 was designed to follow up some participants of the Scottish Mental Survey 1947 – a nationwide general cognitive test carried out among all 1936-born children attending Scottish schools on June 4th, 1947 – with the main aim to study non-pathological cognitive ageing in later life [32, 33]. Surviving participants of the Scottish Mental Survey 1947 living in the City of Edinburgh and the surrounding areas were traced and contacted [33]. Between 2004 and 2007 (Wave 1; mean age = 70 years), 1091 subjects underwent assessments; since Wave 2 (2007−2010; mean age = 73 years; *n* = 866), neuroimaging data are also available [33]. In 2014 (mean age = 78 years), a ‘life grid’ questionnaire was administered among surviving LBC1936 participants to collect retrospective account of their residential history for every decade from birth to date of completion [32]. Flashbulb memory prompts (e.g., 9/11 attacks in New York) and participant supplied personal events assisted recall [29]. Out of 704 contacted cohort members, 593 provided life-course addresses (84% completion rate) which were geocoded using automatic geocoders and historical building databases (see ref. [29] for more detail).

### Neighbourhood deprivation

Life-course neighbourhood deprivation was operationalised as small area-level social disadvantage, computed once for every decade of the study with data derived from administrative records [29]. Due to a lack of consistently measured data for the entire study period we utilised two different indices; limited availability of historical spatial information constrained us to focus solely on the City of Edinburgh. In 1941, 1951, 1961 and 1971, information on overcrowding, population density, infant mortality, tenure, and amenities contributed to an index of multiple deprivation [29]; for 1981, 1991 and 2001, we utilised the Carstairs index of deprivation (i.e., male unemployment, overcrowding, car ownership, low social class) [34]. The two indices were strongly correlated in 1981 (*r* = 0.86), when data for both were available. Geographic data were aggregated to a common spatial resolution (1961 ward-level; *n* = 23) in order to support estimating of missing indicators; *z*-scores were calculated to ensure comparability across the two indices [29].

Geocoded residential addresses (latitude, longitude) were first linked to deprivation scores using 10-year intervals (e.g., 1941 score linked to all 1936−1945 addresses); if there was more than one address in a decade, linked deprivation scores were averaged. Decade-specific neighbourhood deprivation scores closer in time were highly correlated (*r* > 0.61) (Supplementary Fig. 1). Then, we computed three periods capturing average exposures to neighbourhood deprivation during childhood (1936−1955; age ≤19 years), young adulthood (1956−1975; ages 20–39 years) and mid- to late adulthood (1976−2005; ages 40–69 years) for participants having any valid exposure data during these epochs [10]. Accumulated neighbourhood disadvantage was calculated as the mean exposure across the three periods, requiring valid measurement of neighbourhood deprivation (i.e., living in the City of Edinburgh) at least once within each exposure period.

### MRI acquisition

All brain data were acquired between 2007 and 2010 (Wave 2); the magnetic resonance imaging (MRI) acquisition parameters have been described previously [35]. All participants underwent brain MRI on a 1.5 T GE Signa Horizon HDx clinical scanner (General Electric, Milwaukee, WI) with a manufacturer supplied 8-channel phased-array head coil. High resolution 3D T_1_-weighted inversion-recovery prepared, fast spoiled gradient-echo volumes were acquired in the coronal plane with 160 contiguous 1.3 mm thick slices resulting in voxel dimensions of 1 × 1 × 1.3 mm. T_2_-weighted fast spin echo volumes were acquired in the coronal plane with 80 contiguous 2 mm thick slices resulting in voxel dimensions of 1 × 1 × 2 mm. For the diffusion MRI protocol, single-shot spin-echo echo-planar (EP) diffusion-weighted whole-brain volumes (*b* = 1000 s mm^−2^) were acquired in 64 noncollinear directions, along with seven T_2_-weighted volumes (*b* = 0 s mm^−2^). Seventy-two contiguous axial 2 mm thick slices were acquired resulting in 2 mm isotropic voxels.

### Image processing

We assessed both global and regional brain measures. From the T_1_- and T_2_-weighted data, various tissue volumes were estimated as described previously [35]. Total brain volume was estimated as intracranial volume minus cerebrospinal fluid, and grey matter volume as total brain volume minus white matter volume. White matter volume was segmented into normal appearing-white matter and white matter hyperintensities, the latter defined as hyperintense areas (>3 mm in diameter) in white matter. Additionally, cortical reconstruction was performed with the FreeSurfer image analysis suite (http://surfer.nmr.mgh.harvard.edu) v5.1.0. Cortical surface analyses were then performed using the SurfStat MATLAB toolbox (http://www.math.mcgill.ca/keith/surfstat). Surfaces were aligned vertex-wise into a common space (the FreeSurfer average template) and spatially smoothed at 20 mm full width at half maximum, allowing sample-wide analyses of volume, area, and thickness across the cortex.

Diffusion MRI (dMRI) can quantify water molecule diffusion in white matter microstructure [36]. All raw dMRI data were converted from DICOM to NIfTI-1 format using TractoR v2.6.2 [37]. Using tools freely available in the FSL toolkit v4.1.9 (FMRIB, Oxford University: http://www.fmrib.ox.ac.uk) [38], data underwent brain extraction [39] performed on the T_2_-weighted EP volumes acquired along with the dMRI data. The brain mask was applied to all volumes after correcting for systematic eddy-current induced imaging distortions and bulk patient motion using affine registration to the first T_2_-weighted EP volume of each participant [40]. For all dMRI volumes, diffusion tensors were fitted at each voxel and water diffusion measures were estimated for mean diffusivity and fractional anisotropy at each voxel. Tractography was performed using an established probabilistic algorithm with a two-fibre model per voxel (BEDPOSTX/ProbtrackX) [41, 42]. Analysis of twelve major white matter tracts was performed using probabilistic neighbourhood tractography [37]. These tracts were the genu and splenium of the corpus callosum, left and right arcuate fasciculus, left and right anterior thalamic radiation, left and right rostral cingulum, left and right inferior longitudinal fasciculus, and left and right uncinate fasciculus (see Supplementary Fig. 2 for their locations). All tracts were visually quality checked, and exclusions were made on a tract basis. Tract-averaged diffusion parameters (i.e., fractional anisotropy, mean diffusivity) weighted by the streamline visitation count were then calculated from all voxels by tract [35, 43].

### Covariates

**Fig. 1:**
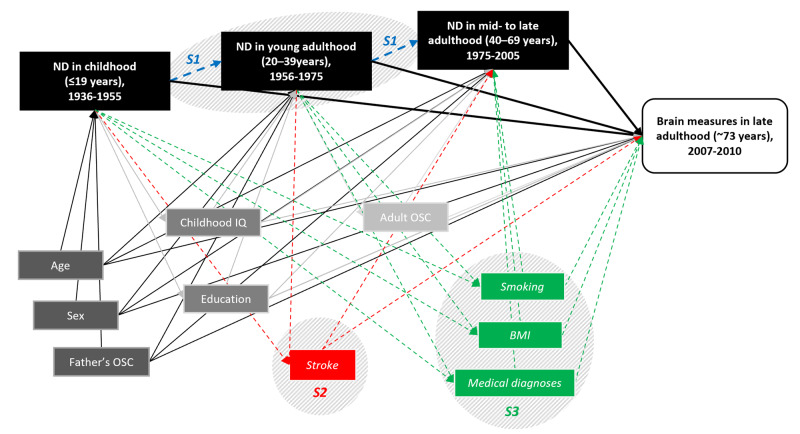
Directed acyclic graph representing associations between neighbourhood deprivation, brain measures and confounders considered in the main and sensitivity analyses. Confounders included in the main analyses are coloured grey: dark grey are considered as confounders for all life course models, medium grey for young adulthood and mid- to late adulthood exposures, light grey for mid- to late adulthood exposures only. Sensitivity analyses addressing selective mobility (S1) and potential confounding (S2, S3) are blue, red, and green, respectively. Links between confounders are not shown for simplicity. BMI = body mass index; IQ = intelligence quotient; ND = neighbourhood deprivation; OSC = occupational social class.

Relevant confounders were selected based on the literature [11, 24] and are presented in a directed acyclic graph (Fig. 1). Age and sex (female, male) were included in all presented models. Father’s occupational social class was classified into high (I/II: professional-managerial) and low classes (III/IV/V: skilled, partly skilled, and unskilled) [44]; the same categorisation was used for own social class in adulthood (for women, husband’s class was taken if higher). Childhood IQ was measured at age 11 years with the Moray House Test No 12 [32], and education was captured as years spent in full-time education. In sensitivity analyses, we considered a range of health-related variables, which can be theorised as confounders, but also as mediators between neighbourhood deprivation and brain health [11]. They were collected at the time of MRI acquisition (i.e., Wave 2) and included stroke identified from MRI scans by a consultant neuroradiologist (yes, no), body mass index (BMI), smoking status (current smoker, past smoker, never smoked), and self-reported medical diagnosis (yes, no) of stroke, diabetes, hypertension, and cardiovascular disease.

### Statistical analysis

Models were fitted with full information maximum likelihood (FIML) estimation within structural equation modelling (SEM) using the *lavaan* package v0.6-12 [45] in R v4.2.1 [46]; codes used in this study are available from the corresponding authors upon request. FIML regression has the advantage of estimating model parameters based on all available information, including participants with missing variables, increasing power, and thus lowering type II error. Importantly, fitting models in the context of all available data for confounders enables to calculate model residuals in a larger and more comprehensive sample, thus estimating the impact of exposure more accurately. FIML regression produces equivalent results to models handling missing data with multiple imputation [47]. In addition to standardised regression coefficients (*β*), standard errors (SE), and two-sided *p*-values (*p*) adjusted for false discovery rate (*p*_*FDR*_), we reported total sample size for each analysis (*N*) and the number of pairwise complete observations (*n*) for associations of interest.

We considered two sets of model adjustment. Model 1 included age and sex. Macro-structural global measures were also corrected for the premorbid brain size by adjusting for intracranial volume; adjustment for premorbid brain size was not applied for diffusion properties as they are not contingent upon anthropometry. Model 2 additionally adjusted for confounders picked individually for each life-course model using the directed acyclic graph (Fig. 1). Father’s social class was confounder for all life-course models, childhood IQ, and education for young adulthood, mid- to late adulthood and accumulation models, while adult occupational social class for mid- to late adulthood and accumulation models. Analyses for global and local brain measures were performed with both Model 1 and Model 2 adjustments.

*Global brain measures* included six macro-structural outcomes (total brain, grey matter, normal-appearing white matter, and white matter hyperintensity [log-transformed to approximate normal distribution [43]] volumes, cortical surface area, and mean cortical thickness) and the two markers of white matter microstructure. Consistent with prior work [48], general factors of fractional anisotropy and mean diffusivity across the twelve white matter tracts were estimated as latent factors; we included residual correlations between the splenium and the genu of corpus callosum, and between right and left sides of the bilateral tracts (see Supplementary Table 1 for fit indices and factor loadings). We corrected for multiple comparison between the eight global brain outcomes using FDR adjustment [49]. Goodness of fit indices were provided for the general factors of fractional anisotropy and mean diffusivity, for all other outcomes models were fully saturated (i.e., Comparative Fit Index=1, Tucker-Lewis Index=1, Root Mean Square Error of Approximation=0, Standardised Root Mean Square Residual=0).

*Local brain associations* were explored in further analyses. First, we estimated associations with life-course neighbourhood deprivation scores across the entire cortical surface. Vertex-wise analysis was performed in a common space (the FreeSurfer average template) for 327,684 cortical vertices using all MRI participants with cortical surface data. Three vertex-wise brain measures were assessed: cortical volume, surface area, and thickness. Vertex measures were exported from SurfStat and then SEM with FIML was used to iteratively estimate standardised coefficients and the corresponding *p*-values by vertex for each neighbourhood deprivation exposure. Correction for multiple comparison was performed by FDR and the findings were presented in cortical surface maps. The spatial overlap between significant cortical regions was assessed by the Dice coefficient [50]. Second, in addition to the general factors, we presented associations in each of the twelve white matter tracts after FDR correction.

*Exploratory and sensitivity analysis* were estimated for global brain measures only and using Model 2 adjustments (if applicable). We performed exploratory analyses to test whether the associations between neighbourhood deprivation and global brain structure differed by sex, father’s, and own adult social class, as well as by *APOE* ε4 allele status (ε4 carriers, not ε4 carriers), which is a genetic risk factor of cognitive decline [51]. Models were fitted with interaction terms; population groups with FDR-significant differences were carried forward to multi-group SEM analysis. Seven sensitivity analyses tested the robustness of associations with global brain measures. First, to address high correlation between neighbourhood deprivation scores across the three epochs, we regressed young adulthood on childhood scores, mid- to late adulthood on young adulthood scores within SEM by preserving their temporal ordering (S1). Second, we adjusted all models for stroke identified from MRI scans; although these were predominantly asymptomatic or mild strokes, they might have had a small effect on brain measures (S2). Third, mid- to late adulthood and accumulation models were further adjusted for health-related variables (i.e., BMI, smoking status, self-reported medical diagnoses) (S3). For sensitivity analyses S1 and S3, we presented % change in standardised coefficients to aid comparison with the main model (i.e., Model 2). Fourth, to reduce bias from recall inaccuracy during residential history recollection we excluded participants with cognitive impairment [10], identified as either reporting dementia or scoring <24 at the Mini-Mental State Examination in any of the available LBC1936 follow-up waves (Waves 1–5) (S4). Fifth, we applied a strict criterion for computing life-course exposures: participants were required to have valid neighbourhood deprivation scores (i.e., live in the City of Edinburgh) for each decade within a sensitive period, and for each decade during their lives for the accumulated deprivation model (S5). Sixth, we presented main analysis with traditionally performed linear regression using complete cases (S6). Finally, we operationalised neighbourhood deprivation as a dichotomous variable with low [bottom 66.6%] versus high [top 33.3%] deprivation (S7).

## Results

There were 689 individuals with at least one global brain measure available; the total sample size was determined by the outcomes and slightly varied across the eight global brain measures (Table 1). The average age was 72.68 years at the time of MRI acquisition, 52.69% of participants were male. Neighbourhood deprivation could be only linked to participants residing in Edinburgh, with the number of pairwise complete observations between each outcome and exposure constellation providing the base for the associations of interest: childhood, young adulthood, mid- to late adulthood, and accumulated neighbourhood deprivation scores were available for a maximum of 316, 388, 400, and 285 participants, respectively. Average deprivation scores decreased across participants’ lives (Table 1), but they remained positively intercorrelated (*r* = 0.26−0.57) (Supplementary Table 2). Individuals living in more deprived neighbourhoods throughout their life-course were more likely to belong to lower occupational social classes in childhood and adulthood (suggesting social segregation by geography and restricted social mobility), had lower childhood IQ scores, and spent fewer years in full-time education (Supplementary Table 3).

**Table 1:** Characteristics of study participants included in the analyses.

Characteristics	Total number	Mean ± SD/number (%)
Global brain measures, mean ± SD^a^		
Total brain volume (cm^3^)	658	988.97 ± 89.44
Grey matter volume (cm^3^)	658	471.56 ± 44.71
Normal-appearing white matter volume (cm^3^)	658	475.01 ± 50.66
White matter hyperintensity volume (cm^3^)	672	12.06 ± 12.84
Surface area (cm^2^)	636	1533.72 ± 144.92
Mean cortical thickness (mm)	636	2.26 ± 0.10
General fractional anisotropy (standardised unit)^b^	665	0 ± 1
General mean diffusivity (standardised unit)^b^	665	0 ± 1
Neighbourhood deprivation, mean ± SD		
In childhood (age 0−19 years)	316	0.57 ± 3.39
In young adulthood (age 20−39 years)	388	−0.85 ± 2.80
In mid- to late adulthood (age 40−69 years)	400	−2.35 ± 2.74
Accumulated (age 0−69 years)	285	−2.00 ± 6.84
Intracranial volume (cm^3^), mean ± SD	680	1450.83 ± 140.52
Age in years, mean ± SD	689	72.68 ± 0.73
Sex, number (%)	689	
Male		363 (52.69%)
Female		326 (47.31%)
Father’s occupational social class, number (%)	629	
High (professional-managerial)		162 (25.76%)
Low (skilled, partly skilled, and unskilled)		467 (74.24%)
Childhood IQ, mean ± SD	652	100.80 (15.30)
Years spent in education, mean ± SD	689	10.80 (1.14)
Adult occupational social class, number (%)	678	
High (professional-managerial)		392 (57.82%)
Low (skilled, partly skilled, and unskilled)		286 (42.18%)
*APOE* ε4 allele status, number (%)	654	
ε4 carriers		194 (29.66%)
Not ε4 carriers		460 (70.34%)
Stroke, number (%)^c^	684	93 (13.60%)
Smoking status, number (%)	689	
Current smoker		56 (8.13%)
Ex-smoker		310 (44.99%)
Never smoked		323 (46.88%)
BMI, mean ± SD	689	27.92 ± 4.49
Self-reported history of		
Cardiovascular diseases, number (%)	689	187 (27.14%)
Diabetes, number (%)	689	75 (10.89%)
Hypertension, number (%)	689	339 (49.20%)
Stroke, number (%)	689	48 (6.97%)

^a^The total number of outcome observations defined the sample size (*N*) of the analyses; specific associations were based on the number of pairwise complete observations (*n*) for the respective covariate-outcome pairs.

^b^Operationalised as latent variable; in this table we report predicted values (standardised unit).

^c^Identified from MRI scans by a consultant neuroradiologist.

### Global brain measures

In the age and sex, (and intracranial volume for macrostructural outcome measures) adjusted model (i.e., Model 1), mid- to late adulthood neighbourhood deprivation was negatively associated with total brain and grey matter volumes, mean cortical thickness, and general fractional anisotropy (*p*_*FDR*_ < 0.05) (Table 2). These associations remained FDR significant after further controlling for relevant life-course confounders (i.e., Model 2): neighbourhood deprivation in mid- to late adulthood was linked to smaller total brain (*β* = −0.06; SE = 0.02; *p*_*FDR*_ = 0.008; *N* = 658; *n* = 390) and smaller grey matter volumes (*β* = −0.11; SE = 0.03; *p*_*FDR*_ = 0.003; *N* = 658; *n* = 390), thinner cortex (*β* = −0.14; SE = 0.06; *p*_*FDR*_ = 0.03; *N* = 636, *n* = 379), and lower general fractional anisotropy (*β* = −0.19; SE = 0.06; *p*_*FDR*_ = 0.006; *N* = 665, *n* = 388) (Table 2; Supplementary Table 4). In addition, in Model 2 there were FDR-significant negative associations between accumulated neighbourhood deprivation and total brain (*β* = −0.06; SE = 0.02; *p*_*FDR*_ = 0.046; *N* = 658, *n* = 276) and grey matter volumes (*β* = −0.10; SE = 0.04; *p*_*FDR*_ = 0.046; *N* = 658, *n* = 276), as well as between mid- to late adulthood neighbourhood deprivation and normal-appearing white matter volume (*β* = −0.07; SE = 0.03; *p*_*FDR*_ = 0.04; *N* = 658, *n* = 390). Although there were further significant associations with other life-course models, these did not survive FDR correction.

**Table 2:** Associations between life-course models of neighbourhood deprivation and global brain measures.

	Model 1^a^				Model 2^b^			
	*β*	SE	*p*	*p*_*FDR*_	*β*	SE	*p*	*p*_*FDR*_
*Childhood neighbourhood deprivation*								
Total brain volume, *n* = 305	−0.03	0.02	0.16	0.70	−0.04	0.02	0.05	0.26
Grey matter volume, *n* = 305	−0.04	0.03	0.18	0.70	−0.06	0.03	0.06	0.26
Normal-appearing white matter volume, *n* = 305	0.01	0.03	0.76	0.87	0.01	0.03	0.85	0.85
White matter hyperintensity volume, *n* = 311	−0.01	0.05	0.81	0.87	−0.02	0.05	0.78	0.85
Cortical surface area, *n* = 296	−0.01	0.03	0.68	0.87	−0.01	0.03	0.69	0.85
Mean cortical thickness, *n* = 296	−0.06	0.06	0.33	0.78	−0.07	0.06	0.24	0.58
General fractional anisotropy^c^, *n* = 307	−0.05	0.06	0.39	0.78	−0.07	0.06	0.29	0.58
General mean diffusivity^c^, *n* = 307	−0.01	0.06	0.87	0.87	0.01	0.06	0.83	0.85
*Young adulthood neighbourhood deprivation*								
Total brain volume, *n* = 376	−0.01	0.02	0.60	0.69	−0.02	0.02	0.28	0.37
Grey matter volume, *n* = 376	−0.03	0.03	0.26	0.38	−0.05	0.03	0.11	0.22
Normal-appearing white matter volume, *n* = 376	0.04	0.03	0.21	0.38	0.04	0.03	0.18	0.30
White matter hyperintensity volume, *n* = 383	−0.01	0.05	0.82	0.82	−0.03	0.05	0.50	0.50
Cortical surface area, *n* = 367	−0.04	0.03	0.17	0.38	−0.06	0.03	0.05	0.20
Mean cortical thickness, *n* = 367	−0.14	0.05	0.01	0.06	−0.10	0.05	0.08	0.20
General fractional anisotropy^c^, *n* = 376	−0.12	0.06	0.04	0.15	−0.14	0.06	0.02	0.17
Gneral mean diffusivity^c^, *n* = 376	−0.06	0.06	0.28	0.38	−0.05	0.06	0.38	0.43
*Mid- to late adulthood neighbourhood deprivation*								
Total brain volume, *n* = 390	−**0.05**	**0.02**	**0.010**	**0.02**	−**0.06**	**0.02**	**0.003**	**0.008**
Grey matter volume, *n* = 390	−**0.09**	**0.03**	**0.003**	**0.01**	−**0.11**	**0.03**	**<0.001**	**0.003**
Normal-appearing white matter volume, *n* = 390	−0.06	0.03	0.05	0.08	−**0.07**	**0.03**	**0.02**	**0.04**
White matter hyperintensity volume, *n* = 396	0.04	0.05	0.39	0.45	0.05	0.05	0.35	0.40
Cortical surface area, *n* = 379	−0.03	0.03	0.22	0.29	−0.05	0.03	0.14	0.19
Mean cortical thickness, *n* = 379	−**0.19**	**0.05**	**<0.001**	**0.002**	−**0.14**	**0.06**	**0.013**	**0.03**
General fractional anisotropy^c^, *n* = 388	−**0.14**	**0.05**	**0.008**	**0.02**	−**0.19**	**0.06**	**0.002**	**0.006**
General mean diffusivity^c^, *n* = 388	−0.02	0.06	0.70	0.70	0.03	0.06	0.68	0.68
*Accumulated neighbourhood deprivation*								
Total brain volume, *n* = 276	−0.04	0.02	0.05	0.16	**−0.06**	**0.02**	**0.01**	**0.046**
Grey matter volume, *n* = 276	−0.06	0.03	0.06	0.16	**−0.10**	**0.04**	**0.01**	**0.046**
Normal-appearing white matter volume, *n* = 276	0.00	0.03	0.98	0.98	0.00	0.04	0.96	0.96
White matter hyperintensity volume, *n* = 281	0.00	0.05	0.93	0.98	−0.01	0.06	0.93	0.96
Cortical surface area, *n* = 268	−0.03	0.03	0.34	0.46	−0.04	0.04	0.31	0.50
Mean cortical thickness, *n* = 268	−0.16	0.06	0.01	0.09	−0.10	0.07	0.15	0.30
General fractional anisotropy^c^, *n* = 276	−0.08	0.06	0.19	0.38	−0.13	0.07	0.09	0.23
General mean diffusivity^c^, *n* = 276	−0.07	0.07	0.28	0.45	−0.04	0.08	0.59	0.78

Models were fitted within the structural equation modelling framework applying full information maximum likelihood estimation. Total sample size was *N* = 658 for total brain, grey matter, and normal-appearing white matter volumes, *N* = 672 for white matter hyperintensity volume, *N* = 636 for cortical surface area and mean cortical thickness, and *N* = 665 for general fractional anisotropy and mean diffusivity; information on the numbers of complete exposure-outcome observations (*n*) is presented in the table. Model fit indices for general fractional anisotropy and general mean diffusivity are in Supplementary Table 4. Bold typeface denotes false discovery rate adjusted significance (*p*_*FDR*_ < 0.05). SE = standard error.

^a^Models were adjusted for sex and age (and intracranial volume for macrostructural measures).

^b^Models were adjusted for sex, age, (intracranial volume for macrostructural measures,) and father’s occupational social class. In addition, young adulthood models were adjusted for childhood IQ and years spent in education, and mid- to late adulthood/ accumulation models also for adult occupational social class.

^c^No adjustment for intracranial volume.

### Local brain measures: vertex-wide analysis

**Fig. 2:**
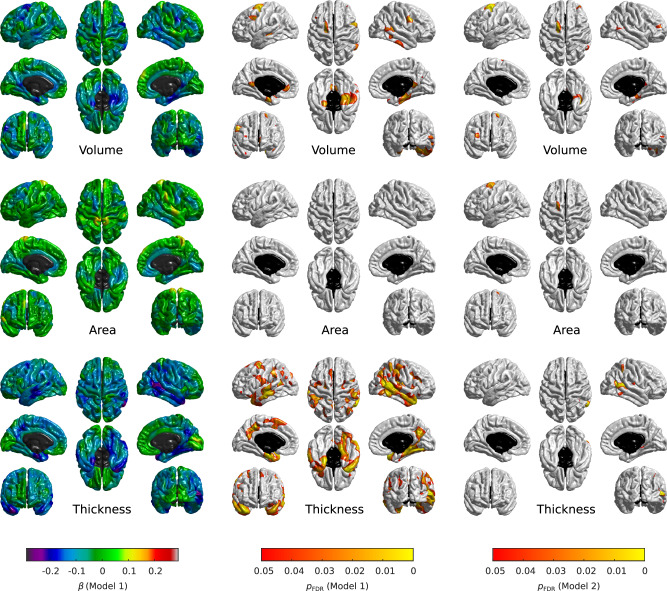
Local associations between mid- to late adulthood neighbourhood deprivation and cortical properties (volume, surface area, and thickness). The fully adjusted standardised coefficients were obtained in linear regression models fitted within the structural equation modelling framework applying full information maximum likelihood estimation. Sample size was *N* = 622, pairwise complete observations were *n* = 371. The heatmaps show (left to right): standardised betas (Model 1), FDR-adjusted *p* values for Model 1 (*p*_*FDR*_ < 0.05), and FDR-adjusted *p*-values for Model 2 (*p*_*FDR*_ < 0.05); the non-cortical mask is shown in black.

We explored local associations across the entire cortical surface using vertex-wise analysis. In Model 1, greater young adulthood and mid- to late adulthood neighbourhood deprivation was associated with lower cortical volume, and a thinner cortex, while greater accumulated neighbourhood deprivation was associated with a thinner cortex (Supplementary Figs. S3–5). After further controlling for relevant life-course confounders (Model 2), the only substantial and statistically significant FDR-corrected associations were for mid- to late adulthood neighbourhood deprivation (Supplementary Figs. S6–8). Specifically, greater mid- to late adulthood neighbourhood deprivation (*N* = 622; *n* = 371) was associated with lower cortical volume (mean *β* = −0.05; *β* range: −0.27 to 0.15); smaller cortical surface area (mean *β* = −0.02; β range: −0.22 to 0.15); and a thinner cortex (mean *β* = −0.07; *β* range: −0.28 to 0.13). There was a spatial overlap in the areas identified across the three cortical measures, particularly between volume and area (Dice coefficient = 0.36), and between volume and thickness (Dice coefficient = 0.10). These significant regions were in the: posterior area of the left superior frontal gyrus (both volume and area); rostral area of the right middle frontal gyrus (volume); right parahippocampal cortex (volume); right isthmus cingulate cortex (volume); caudal area of the right middle temporal gyrus (volume and thickness); caudal area of the right inferior temporal gyrus (thickness); and a lateral area of the right superior parietal lobule (thickness) (Fig. 2). Additionally, there were some small areas of marginal significance between cortical volume and childhood (mean *β* = −0.03; *β* range: −0.25 to 0.16), young adulthood (mean *β* = −0.05; *β* range: −0.24 to 0.09), and accumulated neighbourhood deprivation (mean *β* = −0.05; β range: −0.30 to 0.13) (Supplementary Fig. S6).

### Local brain measures: white matter tracts

**Fig. 3:**
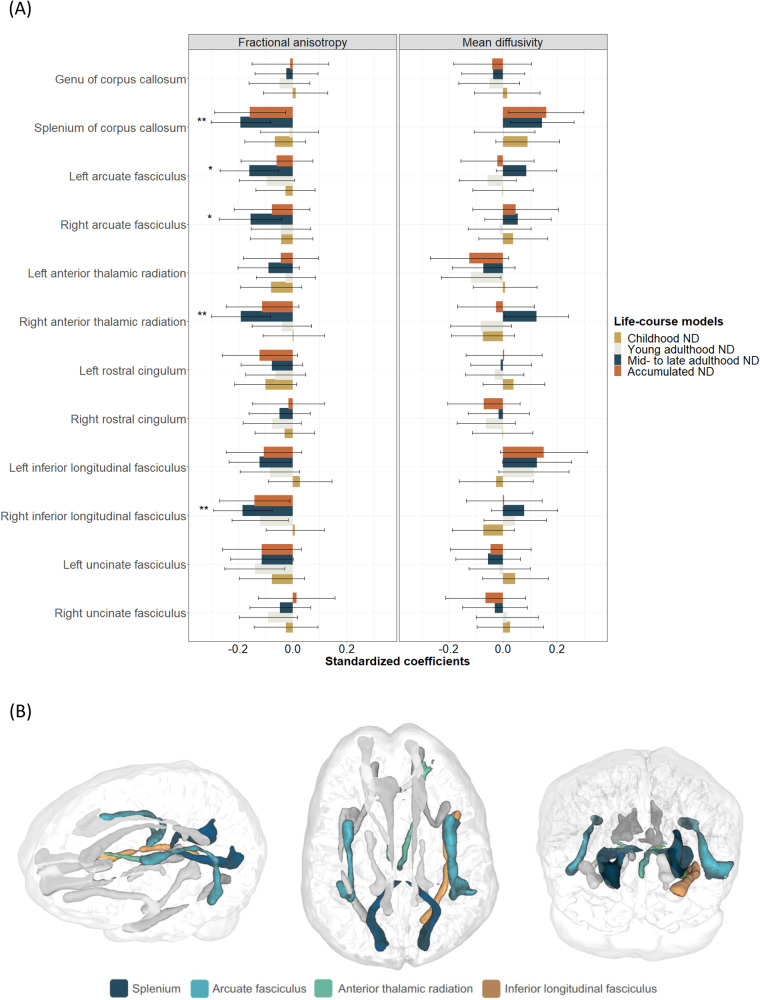
Associations between life-course models of neighbourhood deprivation and white matter microstructure. Associations for fractional anisotropy and mean diffusivity in twelve white matter tracts are shown in Panel **A**, the location of false discovery rate (FDR)-adjusted significant tracts within the brain of a participant in Panel **B**. Standardised coefficients with their 95% CI were obtained from models fitted within the structural equation modelling framework applying full information maximum likelihood estimation. Models were adjusted for sex, age, and father’s occupational social class. In addition, young adulthood models were adjusted for childhood IQ and years spent in education, and mid- to late adulthood/ accumulation models also for adult occupational social class. Asterisks denote FDR-adjusted significance (*p_FDR_ < 0.05; **p_*FDR*_ < 0.01). ND=neighbourhood deprivation.

Associations were also investigated in each of the twelve white matter tracts. After FDR-correction, higher mid- to late adulthood neighbourhood deprivation was associated with lower fractional anisotropy in four tracts, in both Model 1 (Supplementary Table 5) and Model 2 (Supplementary Table 6): splenium of corpus callosum (Model 2: *β* = −0.19; SE = 0.06; *p*_*FDR*_ = 0.003; *N* = 652; *n* = 379), right anterior thalamic radiation (Model 2: *β* = −0.19; SE = 0.06; *p*_*FDR*_ = 0.003; *N* = 662; *n* = 371), right inferior longitudinal fasciculus (Model 2: *β* = −0.18; SE = 0.06; *p*_*FDR*_ = 0.003; *N* = 663; *n* = 386), and left arcuate fasciculus (Model 2: *β* = −0.16; SE = 0.05; *p*_*FDR*_ = 0.01; *N* = 655; *n* = 381). Right arcuate fasciculus became FDR-significant in Model 2 (*β* = −0.16; SE = 0.06; *p*_*FDR*_ = 0.02; *N* = 621; *n* = 360) (Fig. 3). Although there were no significant findings for mean diffusivity, stronger opposite direction associations in the same white matter tracts (e.g., splenium) reinforced findings for fractional anisotropy.

### Social class modifies neighbourhood-brain associations

We explored effect modification by sex, occupational social class in childhood and adulthood, and *APOE* ε4 allele status. No FDR-adjusted differences were detected between females and males, or between ε4 carriers and non-ε4 carriers (Supplementary Table 7). The associations between neighbourhood deprivation and brain structural differences were stronger among individuals belonging to lower occupational social classes in childhood and in adulthood (i.e., skilled, partly skilled, and unskilled). Growing up in relatively disadvantaged households meant stronger negative association between young adulthood neighbourhood deprivation and late adulthood cortical surface area (*β* = −0.12; SE = 0.04; *p*_*FDR*_ = 0.007; *N* = 433; *n* = 255) in comparison to higher social class households (*β* = 0.06; SE = 0.06; *p*_*FDR*_ = 0.50; *N* = 152; *n* = 93).

**Fig. 4:**
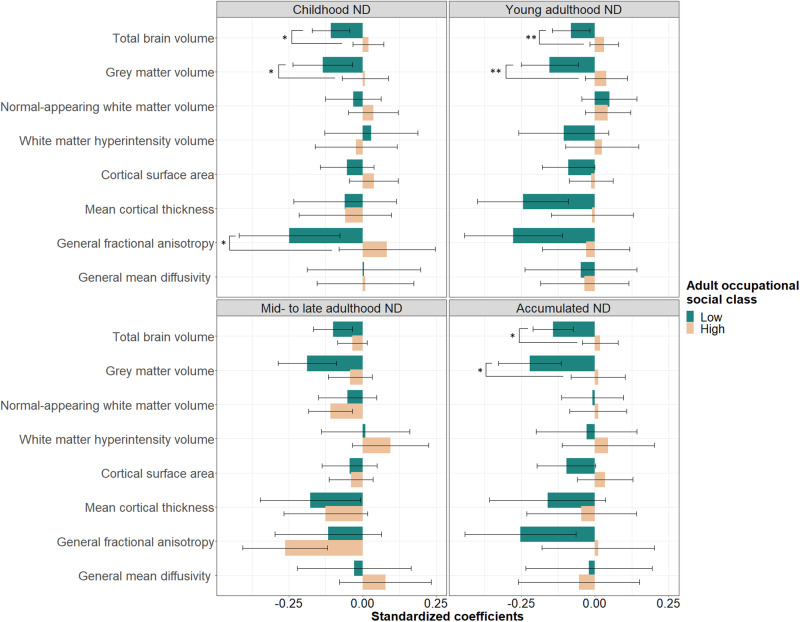
Associations between life-course models of neighbourhood deprivation and global brain measures by occupational social classes in adulthood. High social classes included professional-managerial, low social classes skilled, partly skilled, and unskilled occupations. Standardised coefficients with their 95% CI were obtained from models fitted within the structural equation modelling framework applying full information maximum likelihood estimation. Models were adjusted for sex, age, intracranial volume (for macrostructural measures), and father’s occupational social class; in addition, young adulthood/mid- to late adulthood/ accumulation models were adjusted for childhood IQ and years spent in education. Asterisks denote FDR-adjusted significant interactions (*p_FDR_ < 0.05; **p_*FDR*_ < 0.01). ND=neighbourhood deprivation.

Effect modification was found by own adult social class. In comparison to professional-managerial classes, when participants from lower social classes were exposed to higher childhood, young adulthood, and accumulated neighbourhood deprivation, they had smaller total brain and grey matter volumes, and lower general fractional anisotropy (childhood exposure only). Estimating the associations of accumulated neighbourhood deprivation with total brain (*β* = −0.14; SE = 0.03; *p*_*FDR*_ < 0.001; *N* = 272; *n* = 126) and grey matter volumes (*β* = −0.22; SE = 0.05; *p*_*FDR*_ < 0.001; *N* = 272; *n* = 126) among socially disadvantaged individuals, showed that these were 20% and 14% larger in magnitude, compared to any other life-course models (Supplementary Table 8). Figure 4 depicts associations between life-course models of neighbourhood deprivation and global brain measures by adult occupational social class.

### Sensitivity analyses

*S1*: After regressing young adulthood on childhood, mid- to late adulthood on young adulthood neighbourhood deprivation scores, effect sizes for the global brain measures dropped in magnitude with an average of 9% and 15%, respectively; still, findings remained similar (Supplementary Table 9). *S2*: Adjusting for MRI-identified strokes did not affect the results for mid- to late adulthood exposure; association for accumulated neighbourhood deprivation remained nominally significant (Supplementary Table 10). *S3*: Adjustment for health-related variables reduced effect sizes with 20% and 10% for mid- to late adulthood and accumulated exposures, respectively. Findings remained nominally significant for accumulated exposure, and FDR-corrected significant for mid- to late adulthood neighbourhood deprivation with total brain and grey matter volumes, and general fractional anisotropy (Supplementary Table 11). *S4*: When we excluded individuals from the sample with cognitive impairment, associations remained similar; in addition, young adulthood exposure and general fractional anisotropy, as well as childhood deprivation and total brain volume became FDR-significant (Supplementary Table 12). *S5*: Applying a strict criterion with valid deprivation scores for each decade of the respective life-course model, reinforced the main findings with the exemption for normal-appearing white matter (Supplementary Table 13). *S6*: Utilising linear regression with complete case analysis decreased power, but general fractional anisotropy remained FDR significantly associated with mid-to-late adulthood neighbourhood deprivation, with other nominally significant findings (Supplementary Table 14). S7: Finally, expressing neighbourhood deprivation as a binary variable only found nominally significant associations (childhood exposure with total brain and grey matter volume; young adulthood exposure with cortical surface area and general fractional anisotropy; mid- to late adulthood exposure with grey matter volume and general fractional anisotropy, and accumulated exposure with grey matter volume) (Supplementary Table 15).

## Discussion

This study demonstrates that living in disadvantaged neighbourhoods across the entire life course is linked to brain structural differences among older adults, and this was most consistently found when looking at deprivation in mid-to-late adulthood. First, effects were modest but consistent in their direction across global and local neuroimaging metrics. Of particular note, greater deprivation was most strongly related to a smaller grey matter, thinner brain cortical mantle and to lower white matter fractional anisotropy (a measure of ostensibly ‘poorer’ white matter health, correlated with axonal myelination among other microstructural properties [36]). Specific local associations with both cortical thickness (across medial and lateral temporal and parietal cortex) and with fractional anisotropy of the splenium, anterior thalamic radiation, and in both arcuate and inferior longitudinal fasciculi are notable. These are all regions associated with adult differences in general cognitive ability [30, 52] and with early dementia [53]. Available evidence supports these findings: living in disadvantaged areas is associated with smaller total brain volume [24], and with cortical thinning in Alzheimer’s disease signature regions in older age [26], but not with white matter hyperintensities [25] – in line with our findings. Comparable associations were found in midlife for global brain outcomes [23], for surface area and cortical thickness in regions linked to language and executive functions [22], as well as for white matter fractional anisotropy [21].

Second, mid- to late adulthood neighbourhood deprivation, and to a lesser extent, accumulated neighbourhood deprivation, were linked to brain morphology. While our study is the first to explore the connection between life-course neighbourhood deprivation and brain morphology, related research can provide context. Higher area-level deprivation in later life has been linked to lower cognitive function and faster decline in LBC1936, with early life deprivation’s impact fully mediated through education, occupational social class, and residential mobility [11]. Neuroimaging studies underscore the life-course influence of individual-level socioeconomic position on late adulthood brain health. Swiss research revealed independent correlations between childhood and adulthood socioeconomic position and grey matter volumes in mid- to older adults [9], findings confirmed in an Irish sample, albeit emphasising the predominance of adulthood exposures [7]. In a multicohort investigation, childhood socioeconomic status was also associated with higher risk of lacunes [8]. Similar to our findings, these studies suggest a potentially accumulating life-course impact with stronger associations in more recent exposures.

Third, individual social disadvantage across the life-course amplified the association between neighbourhood deprivation and brain structural differences, with no effect modifications by sex and *APOE* ε4 allele status (aligning with previous studies on neighbourhood exposures [17, 24]). Participants growing up in disadvantaged households showed stronger negative correlation between young adulthood neighbourhood deprivation and cortical surface area. Interaction between family and neighbourhood disadvantage in childhood has been observed in cross-sectional studies, where children from disadvantaged families in more deprived areas show lower cortical thickness [54] and stronger age-related decrease in cortical thickness [55] compared to children from advantaged families. Importantly, childhood neighbourhood deprivation can have long-term consequences for brain development, with longitudinal studies demonstrating lower white matter quantitative anisotropy [56] and greater amygdala reactivity [20] in younger adulthood. Our study adds a life-course perspective to these findings, but also extends previous LBC1936 analysis finding null associations between father’s occupational social class and total brain volume [8]: cooccurring multi-faced disadvantage (individual and area-level) in early life was associated with cortical surface area differences among older adults.

Neighbourhood deprivation was associated with total brain and grey matter volumes only among participants belonging to lower occupational social classes in adulthood, and, for them, across the entire life course: the more time spent living in deprived neighbourhoods, the larger the association between neighbourhood deprivation and brain structural differences. Possibly, through the accumulation model, we may have captured area-level processes affecting the brain’s morphology during both its development in childhood and its degeneration in mid- to late adulthood. Individuals with lower personal resources across the life course, including cognitive reserve [57], were more vulnerable. Alternatively, it is also plausible that in our study, accumulated neighbourhood deprivation only affected the brain during its degeneration phase (e.g., through cumulative allostatic load [58]), with the lagged impact of early life neighbourhood deprivation ‘kicking in’ in later adulthood. However, in the absence of neuroimaging data and area-level deprivation measured across the entire life course within the same individuals, these hypotheses cannot be verified and only present associations without causal implications. As such data are likely many years away from being feasible, future studies should apply pseudo-/accelerated longitudinal designs in different age groups to further explore the reported interactions.

Study strengths included the narrow age-range of participants, the availability of residential history and objectively measured neighbourhood disadvantage covering an exceptionally long period of ~70 years, the detailed assessment of global and local brain measures, and the availability of key life-course social, biological, and psychological confounders. There are also limitations. First, our sample consisted of a relatively healthy, educated, and urbanised group of older adults, with higher childhood IQ [59] and lower risk of mortality [60] in comparison to the population average, leading to selection and survival bias; thus, the homogeneity of our sample presents both a strength and a limitation. Second, reconstructing neighbourhood deprivation indices across the 20th century was challenged by the inconsistent availability of historical data and their varying spatial aggregation in official records. Although 1961 ward geographies likely do not align with participant’s self-defined neighbourhoods, they were necessary to provide a common spatial resolution to handle missing data [29]. Challenges also led to a moderate sample size (ranging from 268 to 396 depending on life-course models), as deprivation scores could be obtained only for individuals residing in Edinburgh. It is plausible that analyses for specific models were relatively underpowered in comparison to others; still, restricting the sample size to participants with observations available for all four models would have led to significant drop in relevant observations (>30%), increased type II error, and sample bias. Given the small effective sample size, we were unable to operationalise some life-course models, such as social mobility; larger studies are required to understand how stable, downward, and upwards residential trajectories associate with brain morphology. Third, residential history was collected in late adulthood. Although residential information is usually recalled with good accuracy [61] and we did not identify bias toward participants with cognitive impairment, we cannot exclude the possibility of retrospective data collection leading to recall bias. Fourth, information on health status prior to late adulthood is not available in LBC1936, which likely increases the risk of unmeasured confounding through selective residential mobility. Fifth, MRI data was only available in late adulthood; therefore, we cannot ascertain whether there is a direct pathway between childhood area disadvantage and later-life brain outcomes, or childhood exposure first influenced childhood brain development. Sixth, as different types of social adversities likely co-occur but influence brain structure differently, disentangling their association with brain structure requires larger and more heterogeneous samples. Last, we tested a large number of associations and, to reduce type 1 error rate, we highlighted only FDR-adjusted significant results. It is plausible that we missed important associations.

## Conclusions

Living in deprived neighbourhoods was associated with markers of poorer brain health among older adults. Neighbourhood deprivation during mid- to late adulthood (age 40–69 years) was associated with brain structural differences in older age, but we also found some evidence for the accumulating impact of neighbourhood deprivation across the entire life course, especially among socially disadvantaged individuals. The life-course approach can provide useful insights into how the social environment might ‘get under the skin,’ and future research should apply it more often to understand differences and changes in brain morphology and related cognition. Greater understanding of relevant brain regions, social and physical neighbourhood features pertinent to brain health, and potential causal pathways require further research attention.

## Supplementary information

Supplementary Material

## Data Availability

The LBCs’ study data have been the subject of many internal (within the University of Edinburgh) and external collaborations, which are encouraged. Those who have interests in outcomes other than cognitive domains are particularly encouraged to collaborate. Both LBC studies have clear data dictionaries which help researchers to discern whether the variables they wish to use are present; these provide a simple short title for each variable, alongside a longer, common-sense description/provenance of each variable. This information is available on the study website (https://www.ed.ac.uk/lothian-birth-cohorts) alongside comprehensive data grids listing all variables collected throughout both LBC studies and the wave at which they were introduced, an ‘LBC Data Request Form’ and example Data Transfer Agreement. Initially, the Data Request Form is e-mailed to the Lothian Birth Cohorts Director Dr Simon R. Cox for approval (via a panel comprising study co-investigators). Instances where approved projects require transfer of data or materials outside the University of Edinburgh require a formal Data Transfer Agreement or Material Transfer Agreement to be established with the host institution. The process is facilitated by a full-time LBC database manager – there is no charge.
